# Tyrosine crystal deposition in pleomorphic adenoma: a rare presentation in a male smoker with long-term follow-up

**DOI:** 10.3332/ecancer.2024.1776

**Published:** 2024-09-23

**Authors:** Sandhya Tamgadge, Gokul Venkateshwar, Treville Pereira, Avinash Tamgadge, Simran Pethani

**Affiliations:** 1 Department of Oral & Maxillofacial Pathology and Microbiology, D.Y. Patil University School of Dentistry, Sector 7, Nerul, Navi Mumbai 400706, Maharashtra, India; 2 Department of Oral & Maxillofacial Surgery, D.Y. Patil University School of Dentistry, Sector 7, Nerul, Navi Mumbai 400706, Maharashtra, India

**Keywords:** adenoma, pleomorphic, tobacco smoking, tyrosine, crystals, minor salivary gland

## Abstract

This case report presents the diagnosis and management of a pleomorphic adenoma in a 55-year-old male smoker with a habit of smoking tobacco for 6 years. The patient presented with a chief complaint of swelling in the posterior palatal region. Clinical examination revealed a well-circumscribed, non-tender, firm swelling in the palatal region. An incisional biopsy followed by surgical excision was performed, and histopathological evaluation confirmed the diagnosis of pleomorphic adenoma. Tyrosine crystals were observed within the tumour stroma, providing additional diagnostic insight. A 10-year post-operative follow-up revealed no recurrence of the lesion.

## Introduction

Pleomorphic adenoma is the most common benign tumour of the salivary glands, characterized by its morphological complexity and diverse histological patterns. This neoplasm derives its name from the architectural pleomorphism seen microscopically, featuring a mix of epithelial and myoepithelial cells arranged in various structural patterns within a mesenchymal-like background [1]. The tumour’s complexity stems from its biphasic nature, demonstrating both epithelial and stromal components, which can vary greatly in proportion and appearance [2]. This case report illustrates this complexity through the observation of multiple cell types (including clear cells, plasmacytoid cells and spindle-shaped cells), the presence of rare tyrosine crystals and areas of osteoid tissue formation within the tumour. Furthermore, the long-term follow-up and discussion of potential etiological factors, such as smoking, contribute to our understanding of the tumour’s biological behavior and risk factors, underscoring the multifaceted nature of pleomorphic adenoma [3]. In this case report, we present a distinctive clinical scenario of a 55-year-old male smoker with a 6-year history of tobacco use. The patient presented with a swelling in the posterior palate, which upon clinical evaluation, was found to be well-defined and non-tender. An incisional biopsy followed by surgical excision confirmed the diagnosis of pleomorphic adenoma. Notably, histopathological examination revealed the presence of tyrosine crystals within the tumour stroma, a rare but significant finding [4]. This observation underscores the importance of meticulous histological analysis in elucidating the nature of salivary gland neoplasms [2, 5]. Furthermore, the 10-year post-operative follow-up, characterized by the absence of recurrence, underscores the efficacy of the chosen management strategy in achieving long-term clinical outcomes [6]. Through this case report, we aim to contribute to the existing literature on salivary gland tumours, emphasizing the relevance of comprehensive diagnostic approaches and tailored therapeutics.

## Case report

**Figure 1.**
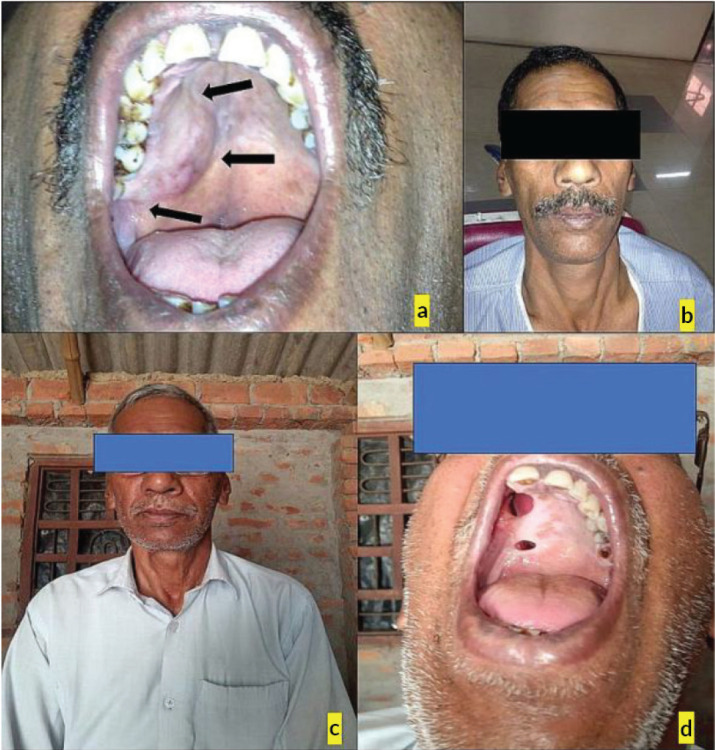
(a): Intraoral photograph, (b and c): Extraoral photograph, (d): Follow-up after 10 years.

A 55-year-old male presented with a swelling in the posterior palatal region, with no relevant medical history aside from a 6-year history of smoking tobacco, and consuming six packets of beedis per day. Clinical examination revealed a well-circumscribed, non-tender, firm swelling extending from tooth 13 to beyond tooth 18, though no swelling was observed extraorally. An incisional biopsy and histopathological evaluation confirmed the diagnosis of pleomorphic adenoma, with the notable finding of tyrosine crystals within the tumour stroma (Figure 1).

Surgical management was performed under general anesthesia using a transoral approach for optimal access to the lesion in the right palate. A full-thickness mucoperiosteal flap was raised from the central incisor to the maxillary right first molar (tooth 18), exposing the underlying hard palate. The lesion was carefully excised in its entirety, ensuring clear margins and minimizing trauma to adjacent tissues. Following the excision, a peripheral ostectomy of the hard palate was performed to remove any potentially involved bone.

**Figure 2.**
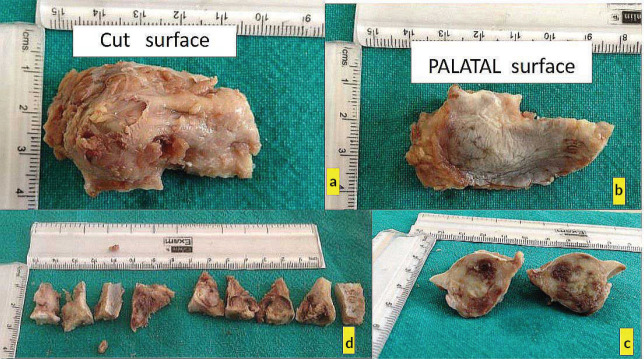
(a–d): Excisional biopsy specimen.

The excised lesion was preserved for histopathological evaluation, and meticulous hemostasis was achieved before the mucoperiosteal flap was repositioned and sutured for proper healing. The patient was provided with detailed postoperative care instructions and scheduled for regular follow-up appointments to monitor healing and evaluate the histopathological findings (Figure 2).

**Figure 3.**
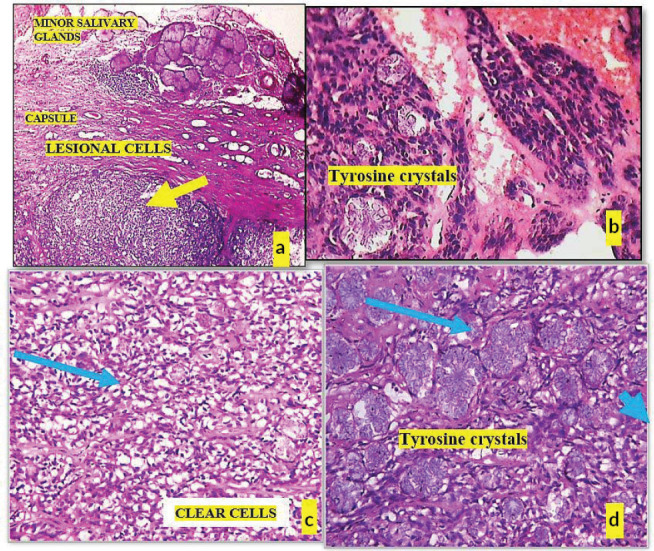
(a): Microphotograph shows lesional tissue (yellow arrow) surrounded by capsule, (b): Tryrosine crystals within lesional tissue, (c): Tumour composed of clear cells (blue arrow) and (d): Plenty of tyrosine crystals are seen (blue arrow).

**Figure 4.**
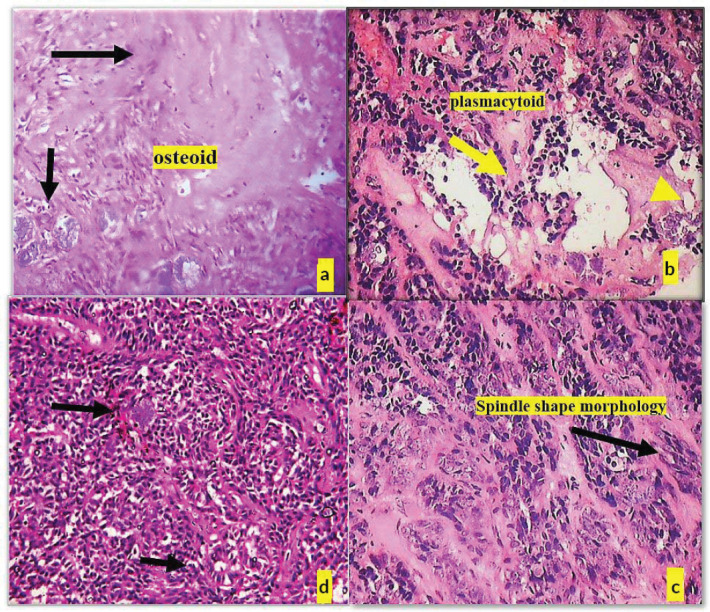
(a): Deposition of osteoid tissue (black arrow) and (b): Cells with plamacytoid morphology (yellow arrow), (c): Clear cell morphology of lesional cells (black arrow), (d): Cells with spindle cells morphology (black arrow).

Histopathological post-surgery evaluation confirmed the diagnosis of pleomorphic adenoma. The specimen showed keratinized epithelium with lesional sheets of cells encased within a capsule and islands of minor salivary gland acini in the surrounding tissue. The lesional cells exhibited a variety of morphological features, including clear, plasmacytoid and spindle-shaped cells, indicative of different myoepithelial cell types. Notably, areas of osteoid tissue added to the histological complexity of the lesion. The presence of tyrosine crystals, dispersed throughout the lesional tissues and capsule in intricate floret-like arrangements, was a striking finding. Additionally, some areas contained luminal structures lined by flat cells resembling endothelial cells (Figures 3 and 4).

A 10-year post-operative follow-up revealed no recurrence of the lesion, indicating successful management. The patient had oroantral fistula for which he has been using a prosthesis.

## Discussion

In this case, report of a 55-year-old male smoker with a 6-year history of tobacco use, the presence of tyrosine crystals within the tumour stroma of the pleomorphic adenoma is a distinctive finding that aided the histological diagnosis, consistent with the observations of Harris and Shipkey [7–9] in 1986.

Myoepithelial cells, with their spindle-shaped morphology and contractile properties, play a key role in the pathogenesis of the lesion. They are integral to salivary gland architecture, contributing to tissue integrity and regulating glandular secretion [10].

The presence of tyrosine-rich crystals in pleomorphic salivary adenoma (PSA) has been a subject of interest in the literature for several decades. Friedmann *et al* [11] were among the first to report these crystals in salivary gland tumours, highlighting their diagnostic significance. Subsequently, Valente *et al* [12] provided a more detailed characterization of these crystalloids using scanning electron microscopy and biochemical analysis, confirming their tyrosine-rich composition.

Margaritescu *et al* [6] conducted a comprehensive study on the tumoural stroma in PSA, including crystalline structures. Their findings enhanced the understanding of the tumour’s microenvironment and the potential role of these crystals in tumour biology.

In the etiopathogenesis of pleomorphic adenoma, it is proposed that genetic alterations or aberrant signalling pathways such as PLAG1 [13, 14] lead to uncontrolled proliferation and abnormal differentiation of myoepithelial cells, resulting in the formation of the characteristic mixed tumour composed of epithelial and myoepithelial components as discussed by various authors [10, 14, 15].

The clinical presentation showed a well-circumscribed, non-tender, firm swelling in the palatal region, extending from tooth 13 to beyond tooth 18. Histopathological examination revealed abundant tyrosine crystals in floret-like arrangements within the lesion, consistent with recent observations by Phulware *et al* [4] in parotid gland pleomorphic adenomas, underscoring the morphological diversity of these structures.

While the exact mechanism behind tyrosine crystal formation remains unclear, it has been postulated to be associated with altered metabolic processes or tissue breakdown as suggested by Eveson and Cawson [16]. The proposed origin involves metabolic dysregulation of tyrosine, a non-essential amino acid, within the tumour microenvironment, leading to its accumulation and crystallization as mentioned by Margaritescu *et al* [6], Phulware *et al* [4]. A detailed structure of tyrosine crystal has been described in the literature by Mostand and Romming [17].

The patient’s smoking history raises questions about smoking’s role in the pathogenesis of salivary gland tumours, as highlighted by several epidemiological studies, including one by Mashberg *et al* [18], which have reported an increased risk of salivary gland neoplasms among smokers, although the underlying mechanisms are not well understood.

The presence of tyrosine-rich crystals is not limited to pleomorphic adenoma. Gould *et al* [9] reported these crystalloids in adenoid cystic carcinoma, while Skálová *et al* [19] observed them in oncocytic cystadenoma of the parotid gland. Bellizzi and Mills [20] described collagenous crystalloids in myoepithelial carcinoma, suggesting a broader significance of crystalline structures in salivary gland pathology.

Tyrosine-rich crystalloids are primarily linked to salivary gland tumours but have also been found in various glandular and non-glandular tumours, including malignant melanoma, chondroid syringoma, vocal cord tumours, nasolabial cysts and salivary gland cysts [21–25]. For instance, Bellizzi and Mills [20] reported collagenous crystalloids in myoepithelial carcinoma, and Reinertsen *et al* [26] observed these structures in a meningioma case. Additionally, Amanda *et al* [22] identified tyrosine crystals in the cutaneous pathology of chondroid syringoma (mixed tumour). It has also been observed in nasolabial cysts in cytology specimen [27]. These observations emphasizing the importance of careful examination and interpretation.

Although the presence of tyrosine crystals in pleomorphic adenomas is linked to specific clinicopathological features, their prognostic implications are still under investigation. Phulware *et al* [4] have explored the potential association between tyrosine crystal formation and favourable outcomes in salivary gland tumours, but further investigations are needed to validate their prognostic utility and elucidate their role in tumour biology.

In our case report, the presence of tyrosine crystals with rare floret-like morphology provided valuable diagnostic insight into the pleomorphic adenoma of a 55-year-old male smoker with palatal swelling. Surgical excision was performed via a transoral approach with a full-thickness mucoperiosteal flap and peripheral ostectomy of the hard palate. A 10-year postoperative follow-up showed no recurrence, supporting the effectiveness of this management approach. This case emphasizes the importance of comprehensive histopathological evaluation and encourages further exploration of the diagnostic and prognostic significance of these crystalline structures in salivary gland pathology [28].

In the context of cytopathology, Carson *et al* [25] and Lemos *et al* [24] reported tyrosine crystals in fine-needle aspirates of benign parotid gland cysts and salivary gland adenomas, respectively. These findings underscore the potential diagnostic value of identifying these crystals in cytological specimens.

## Conclusion

In conclusion, the literature consistently reports the presence of tyrosine-rich crystals in PSA and other salivary gland tumours. Their morphological diversity, as exemplified by our case and recent reports of floret-like structures, adds to the complex histopathological picture of these neoplasms. While their exact biological role remains to be fully elucidated, the consistent observation of these crystals across multiple studies underscores their potential diagnostic and biological significance in salivary gland pathology.

## Conflicts of interest

The authors declare no conflicts of interest.

## Funding

This research did not receive any specific grant from funding agencies in the public, commercial or not-for-profit sectors.
